# Case Report: Chronic inflammatory demyelinating polyradiculoneuropathy rather than hemophagocytic lymphohistiocytosis—the initial phenotype of PRF1 gene mutation

**DOI:** 10.3389/fimmu.2023.1306338

**Published:** 2023-12-11

**Authors:** Lin-Yan Hu, Lin Wan, Qiu-Hong Wang, Xiu-Yu Shi, Yan Meng, Xiao-Fan Yang, Guang Yang, Li-Ping Zou

**Affiliations:** ^1^ Senior Department of Pediatrics, The Seventh Medical Center of Chinese People's Liberation Army General Hospital, Beijing, China; ^2^ Department of Pediatrics, The First Medical Centre, Chinese People's Liberation Army General Hospital, Beijing, China; ^3^ Graduate School, Medical School of Chinese People's Liberation Army, Beijing, China; ^4^ Department of Pediatrics, Shandong University Qilu Hospital, Jinan, Shandong, China

**Keywords:** familial hemophagocytic lymphohistiocytosis, chronic inflammatory demyelinating polyradiculoneuropathy, demyelination of the central nervous system, perforin, perforinopathy

## Abstract

Perforin is essentially involved in the granule-dependent killing activities of cytotoxic T lymphocytes and NK cells. Monoallelic PRF1 mutation increases the risk of autoimmune diseases, and biallelic PRF1 mutation causes familial hemophagocytic lymphohistiocytosis-2. Here, we report a case of a 12-year-old girl with chronic inflammatory demyelinating polyradiculoneuropathy (CIDP), followed by a rapidly progressive onset of hemophagocytic lymphohistiocytosis (HLH) 9 months later, alongside manifestations of demyelinating encephalopathy. Genetic sequencing revealed a heterozygous nonsense mutation in the PRF1 gene (c.984G>A; p.W328*) and a heterozygous missense mutation in the PRF1 gene (c.1349C>T; p.T450M). Eventually, she died because of no suitable allogeneic hematopoietic stem cell available in time. Our observations suggest that CIPD might represent the initial phenotype of biallelic PRF1 mutation and could serve as an early sign of subsequent HLH. A comprehensive understanding of this condition is paramount for timely diagnosis, treatment, and ultimately improved patient outcomes.

## Introduction

The pore-forming protein perforin (PRF1), belonging to the membrane attack complex/PRF (MACPF) protein family, is essentially involved in the granule-dependent killing activities of cytotoxic T lymphocytes (CTLs) and NK cells. Serving as a definite marker of the killing ability of immune cells, PRF1 participates in the establishment of immune homeostasis, elimination of pathogens, and tumor surveillance (1, 2). Biallelic mutations in the PRF1 gene account for up to 30% cases of familial hemophagocytic lymphohistiocytosis (FHL). Monoallelic and biallelic mutations of the PRF1 gene have also been verified to increase the risk of the development of autoimmune diseases, such as autoimmune lymphoproliferative syndrome, type 1 diabetes mellitus, and multiple sclerosis (MS). It has been shown that the frequency of missense/nonsense PRF1 variations is increased in chronic inflammatory demyelinating polyradiculoneuropathy (CIDP) patients, and patients with these variants are more likely to experience relapsing processes and axonal damage (3, 4). Previous studies have identified that three missense mutations of the PRF1 gene, c.272C>T, c.11G>A, and c.1153C>T (resulting in the p.Ala91Val, p.Arg4His, and p.Arg385Trp amino acid substitutions, respectively), and one nonsense mutation, c.1267C>T (leading to the premature stop codon p.Gln423Ter), increased the risk of developing CIDP by 4.47-fold (3). However, the relationship between PRF1 gene variants and CIDP is rarely studied and it is unknown whether patients with CIDP who carry biallelic variants in the PRF1 gene will develop FHL2.

## Case data

A 12-year-old girl initially noticed weakness in her left upper extremity without any inducement. This weakness progressed to affect her left lower extremity and right limb, eventually rendering her unable to walk unassisted. She also experienced atrophy in her left limb, numbness in both hands and lower extremities, and pain in both heels. Notably, she did not report double or blurred vision, blepharoptosis, facial weakness, dysarthria, or shortness of breath. Prior to the onset of her symptoms, she had no significant medical problems. Four months after onset, she was admitted to a local hospital because of her inability to stand. Electromyography demonstrated significantly decreased motor nerve conduction velocity. Head magnetic resonance imaging (MRI), magnetic resonance angiography (MRA), and spinal MRI results revealed no abnormalities. CSF analysis showed normal white blood cells (WBC) and increased protein levels (1,295.3 mg/L). She was diagnosed with CIDP and initiated treatment with intravenous methylprednisolone (20 mg/kg*3 days) and intravenous immunoglobulin (1 g/kg*2 days) pulse therapy, followed by maintenance steroid (2 mg/kg/day), which partially improved her symptoms. However, during the process of oral steroid reduction, CIDP symptoms worsened repeatedly, promoting an increase in oral steroid dosage by her parent. Approximately 9 months after symptom onset, the girl experienced recurrent fever; at the beginning, low fever was not paid attention to, and the peak temperature gradually increased, worsening limb weakness. She was sent to a local hospital again. Blood test results indicated pancytopenia, mild abnormal liver function, slight decrease in Fib, and significantly elevated C-reactive protein, but no specific pathogen was found. As anti-infective therapy was ineffective and her condition deteriorated, she was referred to our hospital.

**Figure 1:**
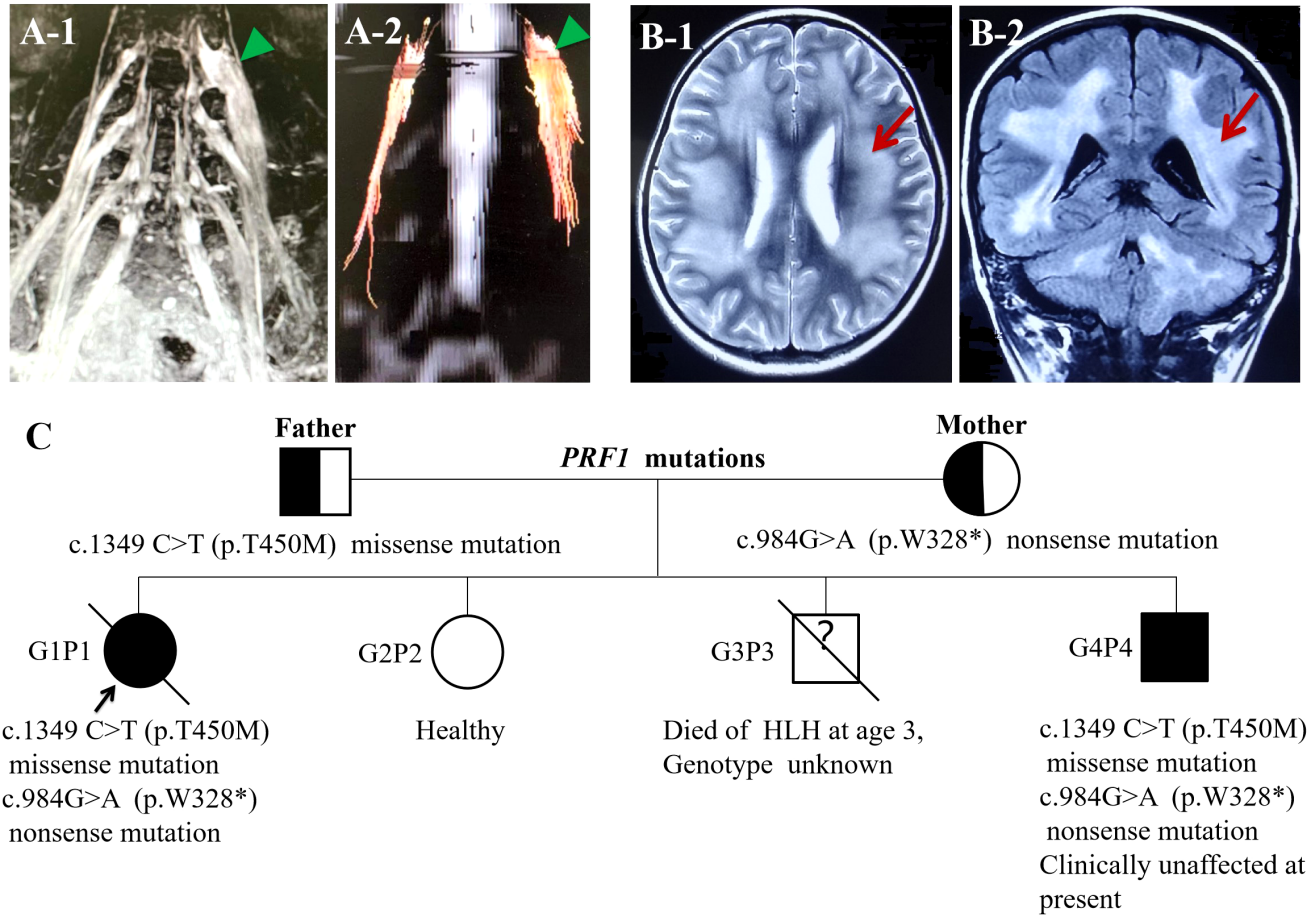
**(A-1)** Lumbosacral plexus MRN: lumbosacral plexus hypertrophy and hypersignal. **(A-2)** Fiber track showed markedly thickened nerve fibers. **(B)** Head MRI: **(B-1)** axial T2WI demonstrated the change of diffused, symmetrical long T2 signal in white matter of cerebral hemispheres. **(B-2)** Coronal FLAIR images revealed the change of generalized, symmetrical high signal in white matter of cerebral hemispheres and cerebellar hemispheres. **(C)** Identified complex heterozygous mutations in the PRF1 gene, c.1349 C>T (p.T450M) (paternal), and c.984>A (p.W328*) (maternal); G3P3 died of HLH at the age of 3 with an unknown genotype; G4P4 carried the same complex heterozygous mutations in the PRF1 gene and clinically unaffected.

Upon admission, her body temperature was up to 39.8°C, and there were two to three heat peaks every day; physical examination demonstrated a pale complexion and hepatosplenomegaly. Neurological examination revealed asymmetrically reduced muscle strength in her limbs, with a Medical Research Council (MRC) score of 3/5 in the left upper limb, 4/5 in the right upper limb, and 2/5 in both lower limbs. Additionally, she exhibited apparent limb atrophy, absent tendon reflexes, impaired perception of temperature, light touch, and pinprick in the limbs, with normal cognition and cranial nerves. Blood test results indicated pancytopenia, increased serum ferritin (>2,000 ng/mL), elevated triglyceride (2.7 mmol/L), and decreased fibrinogen (1.17 g/L). Further tests, including blood culture, EBV DNA, BCG, antinuclear antibody, and anti-ENA antibody showed negative results. The morphology results of medullary cell analysis revealed hemophagocyte. CSF analysis showed normal WBC counts, increased protein levels (1,532.6 mg/L), elevated myelin basic protein (MBP) levels (4.78 nmol/L), and a positive oligoclonal band (OB). Spinal cord MRI results showed abnormal signal in the cervical vertebra and the 3rd lumbar vertebra foramina, extending beyond the spinal canal. Cervical and lumbosacral plexus MRN results revealed extensive enlargement of the brachial plexus trunk and lumbosacral plexus nerve (**Figures 1A-1**). Fiber track results revealed markedly thickened nerve fibers (**Figures 1A-2**). Fluorodeoxyglucose-positron emission tomography/computed tomography (FDG-PET/CT) results also showed an increased 18F-FDG uptake in the thickening plexus nerve, but not elsewhere. Electromyography results exhibited significantly decreased motor nerve conduction velocity (MNCV) and sensory nerve conduction velocity (SNCV) in the bilateral peroneal nerve, tibial nerve, and median nerve. It also revealed a vanished F-wave reflex in the left peroneal nerve and significantly prolonged F-wave latency in the left ulnar nerve (**Table 1**). Based on the patient’s history, clinical manifestations, laboratory findings, and physical examination, a diagnosis of both CIDP and HLH was confirmed.

**Table 1:** The results of electromyography.

Coaxial single-core needle electrode										
Muscle	Voluntary activity	Motor unit potential during light contraction						Motor unit potential during repeated contraction		
		Duration (msec)		Change		Voltage (mV)	Polyphasic	Wave shape		Peak voltage
Left abductor pollicis brevis muscle	–	11.9		↑ 38%		1,781	11%	Simple phase		1.6
Left anterior tibialis muscle	–	13.6		↑ 33%		1,108	–	Simple phase		1.8
Left quadriceps	–	18.8				373	–	Simple phase		1.4
Motor nerve conduction										
Nerve	Latency (msec)				Amplitude (mV)				Conduction velocity (m/s)	
	Proximal		Distal		Proximal		Distal			
Left common peroneal	No response									
Right common peroneal	13.0		4.2		0.3		0.6		26.1	
Left tibial	14.3		4.2		1.5		3.2		29.7	
Right tibial	12.7		3.7		1.6		3.4		33	
Left median	8.7		2.8		3.2		3.4		27.1	
Left ulna	7.6		2.6		4.3		5.0		44	
Sensory nerve conduction										
Nerve	Latency (msec)				Amplitude (µV)				Conduction velocity (m/s)	
Left sural	2.1				7.7				57.1	
Left ulna	2.1				1.5				42.9	
Left median	2.8				1.1				42.9	
F wave										
Nerve	Latency (msec)						Occurrence rate			
Right common peroneal	No response									
Left ulnar	450						15%			

↑, elevated; -, no.

Regarding family history, one of her younger brothers died of HLH at the age of 3. Therefore, primary HLH was suspected, and exon sequencing revealed a heterozygous nonsense variation in the PRF1 gene (c.984G>A; p.W328*) and heterozygous missense variation in the PRF1 gene (c.1349C>T; p.T450M). The protein damage prediction results were analyzed by SIFT, PolyPhen-2, and MutationTaster. The c.1349C>T heterozygous missense variation is harmful and pathogenic, whereas c.984G>A heterozygous nonsense variation is suspected to be pathogenic. The abovementioned mutations might have caused the protein function to be affected. The mutation site c.1349C>T (p.T450M) had been reported in patients affected by FHL2 (https://www.ncbi.nlm.nih.gov/clinvar). While the pathogenicity of the variation c.984G>A has not been reported, nor has it been included in the dbSNP database. None of the abovementioned variations were polymorphic changes, which have an extremely low frequency of occurrence in the population. The compound heterozygous variants found in the PRF1 gene of the patient were inherited from their parents. According to the American College of Medical Genetics (ACMG) guidelines, the above variants might be pathogenic variants that caused the onset of the disease. She was ultimately diagnosed with FHL2. Another 3.5-year-old brother of the patient carried the same complex heterozygous mutations in the PRF1 gene but was clinically unaffected at present (**Figure 1C**).

During hospitalization, she developed demyelinating encephalopathy before the commencement of the HLH-2004 therapeutic regimen, as evidenced by brain MRI, which revealed generalized, symmetrical demyelinating in white matter of the cerebrum and cerebellum hemisphere (**Figures 1B-1, B-2**), and EEG results indicated a slow wave, although subsequent HLH-2004 protocol provided some improvement. The lack of suitable allogeneic hematopoietic stem cells (HSCs) led to disease recurrence and severe lung infections, ultimately resulting in her passing.

## Discussion

FHL caused by the PRF1 gene is common; however, in this case, the patient experienced symptoms of CIPD for 9 months before the onset of HLH. No direct connections between these two diseases have been reported. It is well known that perforin is important for effector functions of cytotoxic T cells and natural killer (NK) cells. Abnormal or absent perforin function because of *PRF1* mutation could lead to impaired killing of target cells, uncontrolled T-cell activation, and high levels of inflammatory cytokines, in turn altering immune system activation and resulting in inflammation and risk of autoimmunity (5).

CIDP is an autoimmune-mediated demyelinating polyneuropathy with chronic progression or remission, resulting from a synergistic interaction of cellular and humoral immune responses. Pathological manifestations include multifocal demyelination of myeloid fibers, endoneuron edema, and inflammatory cell infiltration. Although the etiology and exact pathogenesis remain elusive, clonal expansion of cytotoxic T cells has been observed in the blood and peripheral nerves of patients with CIDP (6, 7). Monoallelic and biallelic pathogenic variations in the PRF1 gene have also been described in patients with CIDP (3, 8). HLH is a multisystem inflammatory disorder; biallelic PRF1 gene pathogenic mutations cause FHL-2, which is characterized by sustained overactivation and excessive proliferation of T lymphocytes and macrophages and increased cytokine levels, leading to infiltration and damage of organs including the bone marrow, liver, and spleen. Thus, we believe that both CIDP and HLH are the result of overactivation of T cells and a cytokine storm.

However, the isolated neurologic manifestations preceding HLH have long been regarded as initial manifestations of primary HLH. A list of child cases that were diagnosed with neurological diseases over the years, including CNS demyelination, MS, intracranial infection, or AIDP, which were later confirmed to have biallelic PRF1 gene pathogenic variations, is summarized in **Table 2** (9–22). In a total of 24 children (including this case), from the onset of neurological symptoms to the final genetic diagnosis, the shortest time was 1 month, the longest time up to 5 years, and one-third of the patients had a brain biopsy. All patients who did not undergo hematopoietic stem cell transplantation (HSCT) died. In fact, it turns out that HLH diagnosis and initiation of treatment have always been delayed. We propose that neuropathy should not be considered a common early-onset symptom of HLH, because the diagnostic criteria for HLH do not encompass the characteristics of autoimmune-mediated neuropathy (23). When the neuropathy, such as CIDP in this case, manifests before HLH, there is a high probability that the diagnosis will be limited to neuropathy, and immunotherapy may mask early signs and symptoms of systemic involvement which can result in a missed optimal treatment window when typical HLH eventually presents (9–22). Early diagnosis is essential for better therapeutic approaches, challenging the existing viewpoint and redefining the relationship between CIDP and HLH. The term “perforinopathy” has been proposed to describe the broad-spectrum manifestations resulting from perforin deficiency caused by abnormalities in the PRF1 gene (24). As in this case, in addition to CIDP, the patient also developed leukoencephalopathy before the commencement of the HLH-2004 therapeutic regimen, and subsequent to treatment with the HLH-2004 protocol, the leukoencephalopathy gradually alleviated. Based on this progression, we attribute both CIDP and leukoencephalopathy to an immune inflammatory damage triggered by the PRF1 gene mutation. HLH represents just one extreme on the spectrum of diseases caused by PRF deficiency. A comprehensive understanding of this concept is crucial for early diagnosis, early treatment, and improved patient prognosis.

**Table 2:** PRF1 gene mutation loci with the nerve system as initial or isolated phenotype and characteristic analysis.

No.	PRF1 gene mutation	Sex	Age at symptom onset	Time to diagnosis of FHL	Diagnostic method	Diagnosis of neurological disorders	Outcome
PRF1 gene mutation loci with CNS as first or isolated phenotype							
1 (9)	c.1376C>T(p.P459L) c.1376C>T(p.P459L)	M	5 ms	7 ms	Gene test	Severe and progressive encephalitis	Died
	c.1376C>T(p.P459L) c.1376C>T(p.P459L)	F	7 ms	26 ms	Gene test	Aseptic meningitis	Died
2 (10)	c.136G>U (p.Glu46stop) c.272C>T (p. Ala91Val) c.355A>U (p.Arg119Trp)	F	3 ys 6 ms	11 ms	Brain biopsy→ gene test	CNS vasculitis ITP	HSCT
3 (11)	c.508A>C(p.S170R) c.727G>T(p. E243X)	F	1 y 6 ms	Around 1–2 ms	Brain biopsy→ gene test	Septic emboli to the brain	HSCT
4 (12)	c.1066C>T(p.R356W) c.1349C>T(p.T450M)	M	16 ys	24 ms	Autopsy→ gene test	Demyelinating disorder	Died
5 (13)	c.673C>T (p.R225W) c.673C>T (p.R225W)	F	12 ys 10 ms	Around 4 ms	Gene test	Brain lymphoma?	HSCT
6 (14)	c.673C>T (p.R225W) c.673C>T (p.R225W)	F	1 y 10.5 ms	38.5 ms	Gene test	Neurodegenerative disorder	Died
	c.673C>T (p.R225W) c.673C>T (p.R225W)	F	6 ys 3.5 ms	1 y 3.5 ms	Gene test	Neurodegenerative disorder	Died
7 (15)	c.394G>T(p.GLY132ARG) c.394G>T(p.GLY132ARG)	M	14 ys	5 ys	Brain biopsy→ gene test	Unknown	HSCT
	c.148G>A(p.Val150MET) c.673C>T (p.R225W)	F	Unknown (saw doctor at the age of 2.5 ys	Unknown	Brain biopsy→ gene test	Encephalitis	Condition deteriorated
8 (16)	c.50delT(p.17fs) c.1229G>C (p.R410P))	F	15 ms	Around 1–2 ms	Gene test	CNS demyelination?	HSCT
9 (17)	c.452A>T (p.H151L) c.666C>A (H222Q)	F	5 ys	60 ms	Brain biopsy→ gene test	Demyelinating CIS CLIPPERS	HSCT
	c.443C>G (p.A148G) c.666C>A (H222Q)	F	6 ys	24 ms	Brain biopsy→ gene test	ADEM MDEM	Relapse occurred 9 months after HSCT
10 (18)	c.634T>C(p.Y212H) c.1083_1094Del(p.361_364del)	F	1 y 5.5 ms	12 ms	Gene test	CNS demyelination	Died
	c.1349C>T(p.T450M) c.853_855del(p.285delK)	F	4 ys 11 ms	14 ms	Gene test	ADEM MS	HSCT
	c.1349C>T(p.T450M) c.1306G>T(p.D436Y)	F	9 ys	37 ms	Gene test	MS	HSCT
	c.65delC(p.22Rfs*2) c.148G>A(p.V50M)	M	1 y 8.5 ms	2 ms	Gene test	CNS demyelination	HSCT
11 (19)	c.4422G>A c.4422G>A	M	5 ys	12 ms	Gene test	ADEM	HSCT
	c.4422G>A c.4422G>A	M	2 ys	1 m	Gene test	Genetic leukodystrophy	HSCT
12 (20)	c.1189_1190dupTG(p.H398Afs*23) c.394G>A(p.G132R)	F	2 ys 7 ms	7 ms	Gene test	Mitochondrial encephalopathy?	HSCT
	c.1189_1190dupTG(p.H398Afs*23) c.394G>A(p.G132R)	M	4 ys 11 ms	10 ms	Gene test	CNS infection; demyelinating disease; metabolic acidosis	Died
13 (21)	c.1519G>T; (p.Glu507Ter)	M	6 ys 5 ms	11ms	Brain biopsy→ gene test	Tuberculous meningitis; CNS vasculitis; MS	Died
	c.1349C>T; (p.Thr450Met)						
PRF1 gene mutation loci with PNS as first or isolated phenotype							
14 (22)	c.694C>T (Arg232Cys) c.1191insTG (His398fsX32)	M	6 ys	1 m	Gene test	AIDP	Died
15	c.1349C>T (p.T450M) c.985G>A (p.W328X)	F	11 ys 3 ms	9 ms	Gene test	CIDP	Died
Other PRF1 gene mutation loci with CIDP as isolated phenotype							
16 (3)	c.272C>T (p. Ala91Val) c.272C>T (p. Ala91Val)	Patients carrying PRF1 variations displayed increased frequency of relapsing forms and axonal damage compared with the other patients. In particular, the presence of PRF1 variations increases the risk of developing relapsing forms by fourfold and axonal damage by 5.3-fold.					
	c.272C>T (p. Ala91Val) c.11G>A (p.Arg4His)						
	c.272C>T (p. Ala91Val) (Monoallelic mutation)						
	c.1153C>T (p.Arg385Trp) (Monoallelic mutation)						
	c.1267C>T (p.Gln423Ter) (Monoallelic mutation)						

ADEM, acute disseminated encephalomyelitis; AIDP, acute inflammatory demyelinating polyradiculoneuropathy; CIDP, chronic inflammatory demyelinating polyradiculoneuropathy; CIS, demyelinating clinically isolated syndrome; CLIPPERS, chronic lymphocytic inflammation with pontine perivascular enhancement responsive to steroids; CNS, central nervous system; HSCT, hematopoietic stem cell transplantation; ITP, immune thrombocytopenic purpura; MDEM multiphasic ADEM; MS, multiple sclerosis; PNS, peripheral nervous system. →, followed by.

Considering the potential association between the genetic etiology and inevitable development of primary HLH, the occurrence of CIDP may serve as an early warning sign. It has been reported that patients with CIDP carrying PRF1 variations exhibit a higher incidence of relapsing forms and increased likelihood of axonal damage compared with those without these variations. The presence of PRF1 gene variations elevates the risk of developing relapsing forms by fourfold and experiencing axonal damage by 5.3-fold. In contrast, no significant difference was detected in terms of gender distribution, disease duration, INCAT disability score, response to first- and second-line treatment, development of dysautonomia, and CNS involvement (3). In our case, the child also manifested axonal injury with a recurrent course. As such, promotion of genetic analysis is recommended for CIDP patients demonstrating recurrent forms and signs of axonal damage. This allows for a window of opportunity for preparation prior to allograft HSCT, including HLA typing and suitable donor searching, thereby avoiding irreversible consequences such as death.

For the patient’s younger brother, who carries the same complex heterozygous PRF1 gene pathogenic mutations but shows no clinical signs of HLH or “perforinopathy”, the molecular analysis was sufficient for an FHL2 diagnosis. He received allogeneic HSCT before the onset of “perforinopathy” and is currently in good health.

## Conclusion

This case highlights the potential for a neurological presentation of “perforinopathy” with CIDP as its initial manifestation. Awareness of this condition is crucial before the onset of HLH, because the disease is treatable. Certainly, for those patients with evidence of a genetic defect, allogeneic HSCT is strongly recommended, as it remains the only curative treatment for primary HLH to date.

## Data availability statement

The raw data supporting the conclusions of this article will be made available by the authors, without undue reservation.

## Ethics statement

The studies involving humans were approved by The Ethics Committee of Chinese PLA General Hospital. The studies were conducted in accordance with the local legislation and institutional requirements. Written informed consent for participation was not required from the participants or the participants’ legal guardians/next of kin in accordance with the national legislation and institutional requirements. Written informed consent was obtained from the minor(s)’ legal guardian/next of kin for the publication of any potentially identifiable images or data included in this article.

## Author contributions

L-YH: Data curation, Formal analysis, Investigation, Writing – original draft. LW: Data curation, Formal analysis, Investigation, Writing – original draft. Q-HW: Data curation, Formal analysis, Investigation, Writing – original draft. X-YS: Supervision, Validation, Writing – review & editing. YM: Supervision, Validation, Writing – review & editing. X-FY: Validation, Writing – review & editing. GY: Conceptualization, Supervision, Validation, Writing – review & editing. L-PZ: Conceptualization, Supervision, Validation, Writing – review & editing.
